# Post Birth Control Syndrome Narratives on TikTok: A Content Analysis

**DOI:** 10.1111/psrh.70026

**Published:** 2025-07-02

**Authors:** Emily J. Pfender, Leah R. Fowler

**Affiliations:** ^1^ Department of OB/GYN University of Pennsylvania Philadelphia Pennsylvania USA; ^2^ University of Houston Law Center Houston Texas USA

**Keywords:** contraception, health communication, post‐birth‐control syndrome, sexual health, social media

## Abstract

**Objective:**

We aimed to examine how TikTok videos tagged with #postbirthcontrolsyndrome (PBCS) made by different types of creators communicate health risks and coping strategies, using the Extended Parallel Process Model (EPPM) to assess threat, fear, and efficacy appeals.

**Methods:**

We conducted a content analysis of the top 100 TikTok videos using the hashtag #postbirthcontrolsyndrome by coding videos for EPPM variables (threat, fear, efficacy), creator type (healthcare provider, content creator, regular user), and engagement metrics (likes, comments, saves).

**Results:**

Content creators frequently portrayed PBCS as severe (40/49) and highlighted side effects (44/49). Healthcare providers emphasized that PBCS is common (13/33) and provided management strategies (14/33; e.g., tips after discontinuing hormonal contraception). Videos describing severe symptoms received higher comments. Preparatory strategy videos garnered more saves.

**Discussion:**

PBCS content on TikTok often uses fear‐based messaging with limited efficacy information, which may influence contraceptive decisions. Differences in framing between creators and providers suggest a need for more evidence‐based communication. Engagement trends indicate that both fear appeals and actionable advice drive more user interaction, reinforcing the importance of balancing emotional salience with credible guidance in social media health messaging.

## 1 Introduction

Individuals on social media are documenting their experiences and symptoms with discontinuing hormonal contraception, including irregular menstrual cycles, hormonal acne, hair loss, mood swings, and digestive issues, and labeling it “post‐birth‐control syndrome” (PBCS) [1]. Contraceptive users believe that PBCS results from the body adjusting to the sudden withdrawal of synthetic hormones [1]. Qualitative research of women who discontinue hormonal contraceptives finds several physical and psychological consequences after discontinuing combined oral contraceptives [2]. While not a recognized clinical diagnosis, naturopathic physicians [3], some medical groups and organizations [1, 3, 4], and TikTok have popularized the idea of PBCS as a syndrome. Some organizations, such as Natural Cycles, have a financial stake in people discontinuing hormonal contraceptives [4].

TikTok is a prominent platform for creators sharing personal health experiences and advice, making it a key space for discussing and normalizing PBCS [5, 6]. Hormonal contraception is a popular topic on social media and includes content about negative side effects and discontinuation [3, 4]. Experimental evidence testing contraceptive messaging found that when viewers perceive TikTok creators to be trustworthy or as having high expertise, they are more likely to intend to use less effective contraception promoted by the creator [6]. Content that encourages discontinuing hormonal contraceptives and emphasizes PBCS side effects could thus discourage individuals from using hormonal contraception, especially among young people who rely on social media for sexual health information [5, 7]. It is also possible that messaging about PBCS could influence individuals to continue using hormonal contraceptives due to fear of discontinuation symptoms despite interest in stopping. The extended parallel process model (EPPM) helps understand the potential impacts of PBCS messages on behavioral intentions regarding contraception.

### 1.1 The Extended Parallel Process Model

Communication scholar Kim Witte developed the EPPM in the early 1990s [8]. Rooted in the field of health communication, the EPPM integrates elements from Protection Motivation Theory and Leventhal's Parallel Process Model [8]. It proposes that individuals assess both the threat (e.g., severity and susceptibility) and their efficacy (e.g., self‐efficacy and response efficacy) in response to a message [8]. The model highlights the importance of balancing threat and efficacy information to elicit a constructive response [8]. It suggests that messages with high perceived threat and efficacy lead to protective behaviors, while messages with high threat and low efficacy result in defensive responses and message rejection [8].

In hormonal contraception discussions, side effects may function as a fear that fuels threat appeals, while tips for managing side effects may function as self‐efficacy. Applying EPPM can help researchers understand how social media messaging can impact hormonal contraceptive discontinuation or continuation decisions and inform sexual education strategies. Thus, our first research question is: To what extent does TikTok content about PBCS present threat, fear, and efficacy appeals?

### 1.2 Messaging by Creator Type

In addition to analyzing how PBCS‐related content aligns with the EPPM framework, it is also important for researchers to consider *who* delivers these messages since the source can significantly influence how audiences receive and interpret them. Research highlights the roles and influences of various social media creators in shaping health‐related perceptions and behaviors [9]. For example, online audiences view healthcare providers as more credible, accurate, and authoritative sources of reproductive health information than those without professional credentials [9]. Outside of healthcare providers, there are other types of creators, including content creators and regular users. Content creators or influencers typically have large followings and monetize their presence, which may motivate them to amplify messages in ways that increase engagement, such as emphasizing fear‐inducing topics to elicit more likes, comments, and views or health benefits from using advertised products [5, 10]. In contrast, regular users often engage with social media primarily for personal expression or community support, without the same incentive structures [5, 10]. Additionally, reproductive health organizations, such as the Guttmacher Institute, contribute evidence‐based health information to these platforms, offering a contrast to both monetized influencer content and anecdotal user posts. Understanding how presentations of PBCS threat, fear, and efficacy messages differ among creator groups may inform how the source of information impacts contraceptive decision‐making and health outcomes. Our second research question is: What are the differences in presentations of PBCS threat, fear, and efficacy for providers, content creators, and regular users?

### 1.3 Engagement With Health Messages on Social Media

Beyond understanding how different creators present PBCS‐related messages, it is also important for researchers to examine how audiences interact with this content, as engagement can reflect the salience and perceived relevance of health messages. Health content on social media with high engagement can reach larger audiences and have greater consequences [11]. Engagement refers to interactions, such as likes, comments, and saves [12]. High engagement suggests the content captures attention and interest [13]. Specific types of health messages receive greater engagement. One study found that social media users were more likely to repost messages with negative appeals than positive appeals [14]. Additionally, social media posts containing messages about the severity of health concerns receive significantly more likes, while self‐efficacy and response efficacy health messages receive significantly fewer [15]. Understanding engagement is important because messages with greater engagement can impact behavioral intentions [11]. Accordingly, our final research question is: Which TikTok videos about PBCS (i.e., threat, fear, efficacy) have the greatest engagement?

## 2 Methods

### 2.1 Sample Selection

On May 13, 2024, EP created a new TikTok account for a 22‐year‐old United States (US) user and retrieved the top 100 videos with #postbirthcontrolsyndrome using TikTok's desktop version, which the research team included in the final sample. According to TikTok's algorithm and hashtag search feature, these were the “top videos” for the selected hashtag that day. Top videos receive high engagement, go viral, or are highly relevant to the search [16]. Additionally, hashtags group topics and allow researchers to pull relevant content. Our team collected the videos on a single day to ensure consistency in the dataset and to capture a snapshot of the most visible and engaged‐with content at that moment in time. Given the dynamic nature of TikTok's algorithm, identifying all videos in the final sample on one day allowed us to minimize variability in content exposure due to algorithmic shifts and ensure that we evaluated all videos under the same conditions. EP also recorded video links and engagement metrics (likes, comments, saves) in an Excel spreadsheet on the same day. No human subjects participated in the study. We did not remove any videos from the final sample because all videos were unique, communicated in English, and relevant to PBCS.

We used quantitative content analysis to examine TikTok videos related to PBCS. The team guided the analysis with the EPPM and applied predefined coding categories for threat, fear, and efficacy appeals. We developed a codebook based on these theoretical constructs and systematically coded content across all 100 videos. While creating the codebook and reviewing sample videos, we identified specific elements related to the severity of PBCS, perceived susceptibility to those side effects, and strategies for managing them. The study team created all codes deductively and added new codes if they emerged. During analysis, we quantified the presence and frequency of these elements. Researchers commonly use quantitative content analysis to study social media [17, 18]. The following section details our codes and coding procedure.

### 2.2 Coding Categories

#### 2.2.1 Creator Type

We categorized creator type by credentials and follower count. We coded content creators as anyone with greater than 10,000 followers. Healthcare providers included those with medical credentials listed in their profile bios, including medical doctors and nurses. We collected credential information during the coding process. We coded healthcare providers with more than 10,000 followers as “healthcare providers who are also content creators.” Regular users included those with less than 10,000 followers and no medical background. Health coaches included those who self‐identified as coaches (e.g., period coach, fitness coach, hormone coach). Organizations included groups or businesses. For analysis, we created three categories: healthcare provider (combined those with > 10k and < 10k followers), content creator, and regular user. There were five types of self‐identified health coaches. Depending on their follower count, the study team collapsed them into content creator or regular user categories. We put health coaches with 10,000+ followers into the content creator group because social media platforms often allow financial incentives when creators reach 10k+ followers (i.e., TikTok Creator Fund) [19]. We put health coaches with less than 10,000 followers into the regular user group because they have few followers and are not medical professionals. Additionally, the sample had only two organizations, and both had more than 10,000 followers, so we moved them into the content creator category.

#### 2.2.2 Previous Contraception Use

We identified whether creators mentioned discontinuing hormonal contraception (i.e., yes/no) and what type of contraception they discontinued (e.g., pill, IUD). We also noted verbal explanations for why they discontinued hormonal contraception (e.g., to improve health). Finally, we coded how long the creator used their hormonal contraception (i.e., less than 1 year, 1 year; did not specify) and the time since discontinuing hormonal contraception (i.e., 1 month, 2–3 months, 4–5 months).

#### 2.2.3 EPPM

To capture perceived threat, we coded susceptibility (i.e., does the creator say PBCS is common; yes/no) and severity (i.e., does the creator say the consequences of PBCS are severe; yes/no). We coded “yes present” for susceptibility when the creator said, “PBCS is common” or “Many people experience PBCS when they discontinue birth control.” We coded “yes present” for severity when the creator said, “Symptoms are extreme, very bad, severe, or intense,” “I'm really going through it,” “I'm suffering,” or “This ruined my life.” We coded for susceptibility and severity because EPPM theory and literature claim that a high perceived threat—comprising both susceptibility and severity—is necessary to motivate protective action.

We coded side effects as fear because side effects serve as tangible consequences that heighten perceived severity, potentially eliciting fear responses. When the creator said they experienced a side effect after discontinuing hormonal contraception, we coded “present” and then selected the specific side effect (e.g., acne, hair loss). If we selected acne as a discussed side effect, we also specified whether the creator showed images of their acne. We assessed efficacy appeals by coding whether the creator provided PBCS management strategies and whether the creator identified PBCS as reversible or permanent.

### 2.3 Coding Protocol

We designed the codebook by individually reviewing #postbirthcontrolsyndrome TikTok videos. To reach consensus on the codes, both members of the study team trained on 50 videos from the same hashtag that were not included in the final sample (i.e., videos after the top 100). After reaching ≥ 0.75 using Gwet's agreement coefficient [20] for all codes in the training sample, both members of the team double‐coded 20% of the final sample, reaching at least ≥ 0.80 reliability [20]. After identifying any discrepancies, we met and rewatched the videos, resolved the discrepancies through discussion, and settled on a final code. One researcher (EP) coded the remaining videos.

## 3 Statistical Analysis

We calculated descriptive statistics for all variables in the codebook. Due to skewed engagement data (i.e., likes, comments, saves), we analyzed relationships using nonparametric Mann–Whitney *U* tests. We conducted all analyses using STATA 17.0.

## 4 Results

Videos averaged 1:04 min, ranging from 0:05 to 5:56 min in length. The median number of likes was 2331.5 (IQR: 11,034). The median number of comments was 58 (IQR: 169.5). The median number of saves was 354 (IQR: 1113). Creators indicated personal experience using hormonal birth control in 86% of videos (68/100). Less than 2% (1/68) of these creators indicated that they spent less than 1 year on hormonal birth control, 18% (12/68) spent more than 1 year on hormonal birth control, and 81% (55/68) indicated prior use of hormonal birth control but did not specify the duration. Table 1 contains descriptive statistics for creator characteristics, the type of contraception discussed, creators' experience using that contraceptive, and time since discontinuation.

**TABLE 1:** TikTok creator characteristics and use of hormonal contraception of top 100 videos with #postbirthcontrolsyndrome collected on May 13, 2024.

	% (*n*)
Creator types	
Content creator	49% (49/100)
Healthcare provider	33% (33/100)
Regular user	18% (18/100)
Discontinued hormonal contraception	68% (68/100)
Type discontinued	
Not specified	69% (69/100)
Pill	26% (26/100)
IUD	4% (4/100)
Nexplanon	1% (1/100)
Reasons for discontinuation	
Not specified	61% (61/100)
Improve health	4% (4/100)
Be more natural	1% (1/100)
Improve energy	1% (1/100)
Improve mood	1% (1/100)
Low sex drive	1% (1/100)
Stop migraines	1% (1/100)
“We can't be on birth control forever”	1% (1/100)
Length of time since discontinuing hormonal contraception	
Less than 1 month	2.9% (2/68)
1 month	2.9% (2/68)
2–3 months	4.4% (3/68)
4–5 months	10.2% (7/68)
6+ months	23.5% (16/68)
Not specified	55.9% (38/68)

For threat appeals, 21% (21/100) of the videos present PBCS as severe, and 22% (22/100) suggest that anyone discontinuing hormonal contraception is susceptible to PBCS. For fear, 84% (84/100) describe negative side effects after discontinuing hormonal contraception. We found 32 negative side effects, and creators mentioned 2.65 (SD = 3.12) side effects on average in each video (Median = 1; IQR: 2). Top side effects included acne (52/84), digestive problems (27/84), and hormone imbalances (24/84). Table 2 includes the top 10 side effects. Of the videos that mentioned acne, 48% (25/52) also showed images of acne. For efficacy appeals, 36% presented ways to improve PBCS (i.e., self‐efficacy; 36/100). The creators mentioned a total of 19 ways to improve PBCS, including taking vitamins (e.g., B vitamins; 12/36) and avoiding processed foods and eating whole foods (10/36). Table 3 includes the top 10 management strategies. For efficacy, 33% (33/100) mentioned that PBCS is reversible, while 9% (9/100) suggested that it may be permanent.

**TABLE 2:** Top 10 side effects associated with PBCS detailed by TikTok creators of top 100 videos with #postbirthcontrolsyndrome collected on May 13, 2024.

Side effects
Acne (52/84)
Digestive problems (27/84)
Hormonal imbalance (24/84)
Depleted nutrients/vitamins (22/84)
Hair loss (19/84)
Mental health issues (15/84)
Amenorrhea (13/84)
Weight gain (11/84)
Headaches/migraines (9/84)
Heavy/painful periods (9/84)

Abbreviation: PBCS = post‐birth control syndrome.

**TABLE 3:** Top 10 management strategies recommended by TikTok creators of top 100 videos with #postbirthcontrolsyndrome collected on May 13, 2024.

Management strategies
Take vitamins (e.g., B, C, D, E) (12/36)
Avoid processed foods/eat whole foods (10/36)
Take magnesium (8/36)
Take probiotic (6/36)
Hire their coaching services (5/36)
Increase protein consumption (5/36)
Take supplements, unspecified (5/36)
Use face wash or skincare routine (5/36)
Consume fermented foods (4/36)
Take folate (4/36)

More content creators said PBCS is severe (40/49, 82%) compared to providers (24/33, 73%) or regular users (1/18, 6%). Additionally, more providers said PBCS is common (13/33, 39%) compared to content creators (8/49, 16%) or regular users (1/18, 6%). Providers (31/33, 94%) and content creators (44/49, 90%) were more likely to mention side effects compared to regular users (9/18, 50%). As for efficacy, more providers (14/33, 42%) listed ways to improve PBCS compared to content creators (18/49, 37%) or regular users (4/18, 22%). More content creators said PBCS is permanent (7/49, 14%) compared to providers (1/33, 3%) or regular users (1/18, 6%).

Results showed no significant difference in comment counts between videos that did and did not highlight PBCS severity (*z* = −0.77, *p* = 0.44). However, severity messages had more comments (Median = 65, IQR: 255) compared to non‐severity messages (Median = 57, IQR: 153). Additionally, there were no significant differences in likes or saves for videos containing perceived severity. Videos containing messages about susceptibility and fear appeals did not have significantly more likes, comments, or saves. There were significant differences for efficacy‐related content. Videos that provided ways to manage PBCS (Median = 719.5, IQR: 2733) received significantly more saves compared to videos that did not (Median = 203.5, IQR: 676) (*z* = −2.64, *p* = 0.01).

## 5 Discussion

This study analyzed TikTok videos tagged with #postbirthcontrolsyndrome to examine how PBCS content aligns with the EPPM, varies across creator types, and engages audiences. Below, the authors discuss key findings related to the three research questions.

First, we found that TikTok messaging that elevates perceived threat and fear, with comparatively limited emphasis on efficacy, dominates content about PBCS. This focus may shape public views on hormonal contraceptives. In the EPPM framework, individuals are more likely to engage in danger control responses, such as seeking preventive measures or behavior change, when they perceive the threat as serious and personally relevant [8]. If perceived threat is low, individuals may disregard the message, whereas if perceived threat is high but efficacy is low, they may engage in fear control responses, such as denial or avoidance [8].

Our findings indicate that fear appeals regarding PBCS side effects are widespread, while efficacy messages that mitigate these fears are less emphasized. In the sample, 84% of videos mentioned negative side effects, with creators describing an average of 2.65 side effects per video. We coded side effects as fear because, according to EPPM, fear arises when individuals perceive harm to their well‐being [8]. Acne, digestive issues, and hormone imbalances were the most commonly cited symptoms, with nearly half of the acne‐related videos also including visual evidence to underscore the severity of the problem. While nearly one‐third of creators suggested PBCS was reversible, only 36% offered strategies to manage symptoms.

Strong fear appeals coupled with limited efficacy messaging may discourage the use of hormonal contraceptives, particularly when viewers do not have tools to mitigate perceived risks. Previous content analysis work finds that creators often use extreme and rare side effects to engage in fearmongering [5, 17], further emphasizing the role of fear appeals in shaping discourse around hormonal contraception. Prior research also suggests that social media may play a role in discouraging the use of hormonal contraceptives more generally [5, 6, 17, 21, 22]. It does not show that exposure to anti‐hormonal content directly influenced attitudes or intentions [6]. However, research has shown that trust in the creator amplifies the effect of their messaging: users are more likely to adopt less effective contraceptive methods if they perceive influencers as credible [6]. While not all viewers will discontinue hormonal contraception due to fear, the EPPM suggests that when threat‐based messaging dominates without corresponding guidance, it may undermine evidence‐based care.

Second, we found differences in presentations of PBCS threat, fear, and efficacy between providers, content creators, and regular users. Content creators were far more likely to describe PBCS as severe (82%) than either healthcare providers (73%) or regular users (6%). Conversely, providers more often framed PBCS as common (39%) and suggested ways to manage it (42%). Only 9% of videos suggested PBCS might be permanent, but most of these came from content creators.

These differences underscore varying perspectives and messaging strategies on social media, influencing viewers' perceptions of contraceptive discontinuation and its health implications. Content creators may rely on emotionally resonant or extreme messaging to boost engagement, including emphasizing worst‐case scenarios. Using emotionally resonant or fear‐inducing messages is particularly relevant when considering the health coaches in the sample, who are not only disseminating health‐related content but also promoting services. Their use of fear‐based messaging may reflect a strategic attempt to heighten perceived risk and motivate viewers to purchase their products. By contrast, providers using social media may be more likely to foreground clinical framing and management strategies. As a result of these competing narratives, audiences may interpret PBCS as more dangerous or irreversible than the available evidence supports.

Finally, the results suggest that videos offering symptom management strategies generated significantly higher engagement—particularly in saves—compared to those that did not. While videos portraying PBCS as severe revealed a similar trend, this difference was not statistically significant. Researchers should further investigate severity messaging in the context of contraception, especially given the non‐normality of the engagement data. While engagement may be partly driven by creator popularity as defined by the number of followers, the EPPM helps explain why these themes resonate: content that heightens fear or offers actionable strategies may prompt stronger emotional reactions, or viewers may perceive them to be more useful.

The study design cannot fully disentangle content effects from creator popularity or algorithmic reach. This limitation is somewhat common in social media research [5, 6, 10, 17, 18]. Nevertheless, the findings hold practical relevance: TikTok's algorithm favors content that generates higher engagement, regardless of the cause of that engagement. The higher save rates for videos offering management strategies and advice underscore many viewers' desire for practical solutions to manage PBCS symptoms, reflecting a real demand for accurate information on symptom prevalence and severity.

Although the medical community does not officially recognize PBCS as a medical diagnosis, it is clearly a source of concern among social media users. Healthcare professionals play an important role in providing evidence‐based guidance [21]. Contraceptive counseling should offer detailed guidance on managing PBCS symptoms to support individuals discontinuing hormonal contraception with confidence. Healthcare providers and public health communicators should also consider participating more actively on platforms like TikTok to share nuanced, empathetic, and evidence‐informed messages about contraception use and discontinuation.

## 6 Limitations and Future Directions

This study has several limitations. First, we limited the sample to TikTok videos retrieved on a single day, which may not fully capture the breadth or evolution of content over time. Findings are specific to TikTok and may not generalize to other platforms with different user demographics, algorithms, or engagement dynamics. By selecting videos using the hashtag #postbirthcontrolsyndrome, we may have excluded relevant content posted under different hashtags or with no hashtags at all. Some videos in the sample did not verbally mention “post‐birth‐control syndrome,” but we included these videos based on hashtag use and topical relevance, introducing some risk of misinterpretation or omission of related content that used different terminology.

Second, we could not control for the number of followers, view counts, or baseline popularity of each account, which may strongly influence engagement metrics such as likes, comments, and saves. TikTok's algorithm does not make all engagement or reach data publicly accessible, limiting the authors' ability to assess the independent effects of message content or to generalize findings about the relative impact of fear or efficacy appeals. Some videos had comments turned off, which may have further affected engagement analysis.

Third, we did not assess the scientific accuracy or credibility of the information presented. Finally, the content analysis only focused on acne as a visual fear appeal. There were very few images of other side effects, often because they are not the type of side effects that can be photographed (e.g., mental health issues or hormonal imbalances) or posted to social media without violating the platform's acceptable use policy (e.g., heavy menstrual periods). However, future research could systematically code visual representations of a broader range of symptoms. A more comprehensive analysis of imagery could deepen the understanding of how visual content contributes to audience perceptions, risk appraisals, and engagement with health‐related messaging.

Finally, we did not quantitatively or qualitatively capture the extent of the seriousness of the side effects. To better align with the implications of threat‐based messaging, an understanding of the severity of each side effect could provide further insights into the study implications.

## 7 Conclusion

PBCS content on social media emphasizes fear‐based messaging, particularly among content creators. Differences in messaging between content creators, healthcare providers, and regular users reveal the competing narratives shaping public perceptions of hormonal contraception. Given the high engagement with both fear and efficacy appeals, public health communicators and healthcare providers should consider more active engagement on social media to counter unreliable narratives and offer balanced, evidence‐based messaging about discontinuing hormonal contraceptives.

## Conflicts of Interest

The authors declare no conflicts of interest.

## Data Availability

The authors are happy to share data and materials upon request.

## References

[1] L. Sharkey , “Everything You Need to Know About Post‐Birth Control Syndrome,” *Healthline*, March 31, 2020, https://www.healthline.com/health/post‐birth‐control‐syndrome.

[2] E. Mu , J. Park , M. Goodman , et al., “Discontinuation of Hormonal Contraceptives and Women's Health: A Scoping Review,” Frontiers in Global Women's Health 3 (2022): 9237896, https://www.ncbi.nlm.nih.gov/pmc/articles/PMC9237896/.

[3] J. Brighton , Beyond the Pill (Harper One, 2019).

[4] Natural Cycles , “Anxiety After Stopping Birth Control: What You Need to Know,” *Natural Cycles*, June 13, 2023, https://www.naturalcycles.com/cyclematters/anxiety‐after‐stopping‐birth‐control.

[5] E. J. Pfender , K. Tsiandoulas , S. Morain , and L. R. Fowler , “Hormonal Contraceptive Side Effects and Nonhormonal Alternatives on TikTok: A Content Analysis,” Health Promotion Practice 26 (2024): 407–411, 10.1177/15248399231221163. 38166482

[6] E. J. Pfender and S. E. Caplan , “The Effect of Social Media Influencer Warranting Cues on Intentions to Use Non‐Hormonal Contraception,” Health Communication 40 (2024): 1428–1442, 10.1080/10410236.2024.2402161. 39258763

[7] Colorado Center for Reproductive Medicine (CCRM) , Women's Health & Post‐Birth Control Syndrome (CCRM, 2023), https://www.ccrmivf.com/news‐events/womens‐health‐post‐bc‐syndrome/.

[8] K. Witte , “Putting the Fear Back Into Fear Appeals: The Extended Parallel Process Model,” Communication Monographs 59, no. 4 (1992): 329–349, 10.1080/03637759209376276.

[9] C. E. Kirkpatrick and L. L. Lawrie , “Can Videos on TikTok Improve Pap Smear Attitudes and Intentions? Effects of Source and Autonomy Support in Short‐Form Health Videos,” Health Communication 39 (2023): 1–2078, 10.1080/10410236.2023.2254962. 37691164

[10] E. J. Pfender , C. Wanzer , and A. Bleakley , “A Content Analysis of Social Media Influencers' ‘What I Eat in a Day’ Vlogs on YouTube,” Health Communication 39, no. 11 (2023): 2244–2255, 10.1080/10410236.2023.2260966. 37743622

[11] R. J. Lee‐Won , K. Na , and K. Coduto , “The Effects of Social Media Virality Metrics, Message Framing, and Perceived Susceptibility on Cancer Screening Intention: The Mediating Role of Fear,” Telematics and Informatics 34, no. 8 (2017): 1387–1397, 10.1016/j.tele.2017.06.002.

[12] M. Trunfio and S. Rossi , “Conceptualising and Measuring Social Media Engagement: A Systematic Literature Review,” Italian Journal of Marketing 2021, no. 3 (2021): 267–292, 10.1007/s43039-021-00035-8.

[13] J. Berger and K. L. Milkman , “Emotion and Virality: What Makes Online Content Go Viral?,” NIM Marketing Intelligence Review 5, no. 1 (2012): 18.

[14] X. Liu , J. Lu , and H. Wang , “When Health Information Meets Social Media: Exploring Virality on Sina Weibo,” Health Communication 32 (2017): 1252–1260, 10.1080/10410236.2016.1217454. 27668831

[15] W. Zou , W. J. Zhang , and L. Tang , “What Do Social Media Influencers Say About Health? A Theory‐Driven Content Analysis of Top Ten Health Influencers' Posts on Sina Weibo,” Journal of Health Communication 26, no. 1 (2021): 1–11, 10.1080/10810730.2020.1865486. 33372857

[16] TikTok , “Popular Creators—Creative Center,” TikTok for Business, accessed April 4, 2025, https://ads.tiktok.com/business/creativecenter/inspiration/popular/creator/pc/en.

[17] E. J. Pfender and M. M. Devlin , “What Do Social Media Influencers Say About Birth Control? A Content Analysis of YouTube Vlogs About Birth Control,” Health Communication 38, no. 14 (2023): 3336–3345, 10.1080/10410236.2022.2149091. 36642835

[18] L. R. Romann and E. J. Pfender , “Disseminating Premenstrual Dysphoric Disorder Information on TikTok: A Content Analysis,” Health Communication (2024): 1–10, 10.1080/10410236.2024.2442685. 39688819

[19] TikTok , “TikTok Creator Fund: Your Questions Answered,” *TikTok Newsroom*, July 2020, https://newsroom.tiktok.com/en‐gb/tiktok‐creator‐fund‐your‐questions‐answered.

[20] K. L. Gwet , Handbook of Interrater Reliability (STATAXIS Publishing Company, 2002).

[21] A. Schneider‐Kamp and J. Takhar , “Interrogating the Pill: Rising Distrust and the Reshaping of Health Risk Perceptions in the Social Media Age,” Social Science & Medicine 331 (2023): 116081, 10.1016/j.socscimed.2023.116081. 37441974

[22] E. J. Pfender and L. R. Fowler , “Social Media Is Influencing Contraceptive Choice,” Journal of Women's Health 33, no. 5 (2024): 563–564, 10.1089/jwh.2023.1152. 38386797

